# Comparative Study on Protein-Rich Electrospun Fibers for In Vitro Applications

**DOI:** 10.3390/polym12081671

**Published:** 2020-07-27

**Authors:** Iriczalli Cruz-Maya, Alessio Varesano, Claudia Vineis, Vincenzo Guarino

**Affiliations:** 1 Institute of Polymers, Composites and, Biomaterials—National Research Council (IPCB-CNR), V.le J.F. Kennedy 54, Mostra d’Oltremare, Pad. 20, 80125 Naples, Italy; cdiriczalli@gmail.com; 2 Intelligent Systems and technologies for advanced manufacturing—National Research Council (STIIMA-CNR), C.so G. Pella 16, 13900 Biella, Italy; alessio.varesano@stiima.cnr.it (A.V.); claudia.vineis@stiima.cnr.it (C.V.)

**Keywords:** keratin, gelatin, zein, electrospinning, cell materials interaction

## Abstract

Electrospinning is the leading technology to fabricate fibrous scaffolds that mimic the architecture of the extracellular matrix of natural tissues. In order to improve the biological response, a consolidated trend involves the blending of synthetic polymers with natural proteins to form protein-rich fibers that include selected biochemical cues able to more actively support in vitro cell interaction. In this study, we compared protein-rich fibers fabricated via electrospinning by the blending of poly ε-caprolactone (PCL) with three different proteins, i.e., gelatin, zein, and keratin, respectively. We demonstrated that the peculiar features of the proteins used significantly influence the morphological properties, in terms of fiber size and distribution. Moreover, keratin drastically enhances the fiber hydrophilicity (water contact angle equal to 44.3° ± 3.9°) with positive effects on cell interaction, as confirmed by the higher proliferation of human mesenchymal stem cells (hMSC) until 7 days. By contrast, gelatin and zein not equally contribute to the fiber wettability (water contact angles equal to 95.2° ± 1.2° and 76.3° ± 4.0°, respectively) due to morphological constraints, i.e., broader fiber diameter distribution ascribable to the non-homogeneous presence of the protein along the fibers, or chemical constrains, i.e., large amount of non-polar amino acids. According to in vitro experimental studies, which included SEM and confocal microscopy analyses and vitality assay, we concluded that keratin is the most promising protein to be combined with PCL for the fabrication of biologically instructive fibers for in vitro applications.

## 1. Introduction

Tissue engineering is a multidisciplinary field that involves the use of polymer technologies to develop smart scaffolds able to promote in vitro regeneration mechanisms [1,2]. The interaction between scaffolds, cells, and biochemical signals is based on the capability to mimic the extracellular matrix (ECM) of native tissues [3,4]. The native ECM provides several functions including (a) support for cells; (b) storage of growth factors; (c) stimulation of cell differentiation; (d) balance of tissue homeostasis; and (e) response in the presence of tissue injury [5]. This mainly depends upon the peculiar architecture of ECM, which is organized in a 3D network of fibrous proteins and polysaccharides assembled in a unique topography that is able to provide biochemical and biophysical signals to cells [6,7].

The challenge to mimic the ECM of tissues is now focused on the investigation of natural materials, to explore how their innate biocompatibility plays a fundamental role in the cell behavior, during the regeneration process [8]. Among them, structural proteins such as gelatin, fibrin, elastin, keratin, silk, and zein are gaining particular interest due to their similarity in terms of molecular structure with collagen, which is abundantly present into all natural tissues [9,10,11]. However, all the structural proteins show some specific chemical/physical features that concur to particularize their interface with cells. For instance, gelatin is the denatured form of collagen derived from the partial hydrolysis of collagen. Depending on the extraction and manufacturing methods, it is commercialized in two forms, obtained after acidic (Type A) and alkaline (Type B) treatments [12], with different content of Arg–Gly–Asp (RGD)-like sequences that relevantly influence the mechanisms of cell adhesion [13]. Keratins show a characteristic secondary structure characterized by intermediate filaments with α-helices containing sulfur content. This confers a globular architecture to the proteins due to the interactions, via disulfide bonds, among sulfur groups and intermediate protein filaments [14]. The presence of cell binding motifs, such as RGD and LDV (Leu–Asp–Val) and their ability to self-assemble, make keratins particularly interesting as natural biomaterials for tissue regeneration [15]. Zein is a vegetable protein found in the endosperm of corn [16,17] by the amino acid sequence characterized by residues i.e., glutamine, alanine, proline, and leucine, with a neutral charge [18]. Due to its peculiar composition, zein is a hydrophobic protein that should inhibit cell attachment; however, it has been widely proposed in drug delivery systems to allow a controllable and sustained release [17,19].

Although the biocompatibility of natural proteins has been variously proved, some specific properties such as instability in water and low mechanical strength still represent relevant constraints for their success in vitro. To improve the fiber stability, the use of chemical compounds has often been considered to promote the formation of additive chemical/physical bonds among the protein chains. However, several studies have reported some criticisms in terms of cytotoxicity—due to the presence of unreacted agents not completely removed—and/or morphological alterations of fiber surfaces, with negative effects on cell interactions [20,21].

An alternative route is currently represented by the blending of proteins with synthetic polymers such as poly ε-(caprolactone) (PCL), poly(glycolic acid) (PGA), poly(lactic acid) (PLA), and their copolymers. Recent literature has demonstrated that this strategy is satisfying to impart biochemical signals to inert/mechanically stable fibers [22,23] with interesting biological outcomes [23,24,25,26]. Indeed, folded chains of proteins strictly embedded with long chains of synthetic polymers allow the formation of building blocks for the fabrication of instructive platforms able to preserve the biochemical properties of proteins (i.e., cell signaling, immune responses, cell adhesion), partially overcoming their intrinsic limitations in terms of functionality loss, due to the fast solubilization in vitro [27].

Herein, a comparative study of electrospun scaffolds made by the blending of PCL with each of three different proteins, i.e., gelatin, zein, or keratin, was assessed. Starting from recent experimental evidence on singularly tested equivalent systems, we aimed at elucidating the contribution of proteins to morphology, chemical properties (i.e., hydrophilicity) and, ultimately, the in vitro response of mesenchymal stem cells, in order to identify the most promising protein-rich fibers suitable for biomedical applications, including scaffolds for interface tissue engineering or coatings of implantable devices.

## 2. Materials and Methods

### 2.1. Preparation of Protein-Rich Fibers

Gelatin (type A from porcine skin, Sigma-Aldrich, Milan, Italy), zein (Sigma-Aldrich, Milan, Italy), keratin (extracted from wool as reported [26], and PCL (M_w_: 65 kDa, Sigma-Aldrich, Milan, Italy) were each dissolved individually in 1,1,1,3,3,3-hexafluoro-2-propanol (HFIP) (Sigma-Aldrich, Italy) for 24 h at room temperature, until a 10% (*w/v*) solution was formed. Then, PCL was mixed in a ratio of 50:50 with gelatin (PCL/gelatin), zein (PCL/zein), and keratin (PCL/keratin) to obtain the blended solutions.

Electrospun fibers were fabricated via electrospinning by the use of automated equipment (NANON 01; MECC, Fukuoka, Japan). The solutions were each ejected by using a plastic syringe (5 mL) connected to a stainless-steel needle. The parameters for the deposition of the fibers were optimized, as indicated in Table 1, at controlled humidity (45%) and temperature (26 °C). As a function of the different properties of proteins used—in terms of polar group—different voltages were used, case by case, to obtain the fibers.

### 2.2. Morphology Characterization

Morphological features of PCL/gelatin, PCL/zein, and PCL/keratin fibers were investigated via field emission scanning electron microscopy (FESEM; Quanta FEG 200 FEI, Eindhoven, The Netherlands), working in high vacuum mode at 5 kV (fixed working distance 100 mm). According to previous studies [28], samples were coated with a Pd–Au nanolayer using a sputtering machine (Emitech K550, Emitech Srl, Corato, Italy) to improve surface conductivity. Average fiber diameter (AVS) was calculated on selected SEM images by the support of open-source image analysis software (ImageJ 1.52i; National Institutes of Health, Bethesda, MD, USA). Results were reported as mean ± standard deviation (SD).

### 2.3. Attenuated Total Reflection Fourier Transform Infrared Spectroscopy Analyses

Fiber composition was basically analyzed by the use of Fourier transform infrared spectroscopy coupled with attenuated total reflectance technique (ATR-FTIR, Perkin Elmer Spectrum 100 FTIR spectrophotometer, Perkin Elmer, Waltham, MA, USA). All the spectra were elaborated into the range between 4000 and 400 cm^−1^ using dedicated software (OriginPro 8 SR0; OriginLab Corporation, Northampton, MA, USA).

### 2.4. Water Contact Angle

Water contact angle analysis was performed with an EasyDrop system (Krüss, Hamburg, Germany) equipped with a Stingray video-camera (Allied Vision Technologies, Ahrensburg, Germany). Water was obtained by Milli-Q (Millipore Corporation, Burlington, MA, USA) with 0.05 μS at 25 °C. Drop volume was fixed to 5 μL with a flow-rate of 180 μL min^−1^. Experimental analyses were carried out at room temperature (23 °C) in the presence of a relative humidity of 63%. Drop Shape Analysis v. 1.92 software was used to measure the contact angle formed after the contact of the drop with the nanofibers, within 3 s. A conic equation was used to fit drop profiles. The derivative of the fitting equation at three-phase contact point was utilized to measure the contact angles. Results were reported as average value of five measurements.

### 2.5. In Vitro Tests

Human mesenchymal stem cells (hMSCs; Sigma-Aldrich, Milan, Italy, SCC034) in the quantity of 5 × 10^3^ were cultured onto PCL/gelatin, PCL/zein, and PCL/keratin nanofibers and incubated in Eagle’s alpha minimum essential medium (α-MEM) supplemented with 10% fetal bovine serum, antibiotic solution (100 μg/mL streptomycin and 100 U/mL), and 2 mM L-glutamine at 37 °C in humidified atmosphere with 5% CO_2_ and 95% air. Specimens were preliminary cut into a disc shape and then sterilized with UV light for 1 h. Cell proliferation was assessed by using cell counting kit-8 (CCK-8, Dojindo, Kumamoto, Japan) to analyze hMSC proliferation at 1, 3, 7, and 14 days. For each experimental time, culture media was refreshed and 10% (*v/v*) of CCK-8 reagent was added. For the analysis, the medium was removed after 4 h and placed into a 96-well plate reader. Absorbance measurements were recorded at 450 nm in a spectrophotometer (Wallac Victor3 1420, PerkinElmer, Boston, MA, USA). Results were represented as mean ± standard deviation and analyzed by Student’s *t*-tests to determine the differences among the groups, considering *p* < 0.05 as statistical significance.

### 2.6. Cell Morphology

hMSC morphology onto protein-rich fibers was evaluated at 24 h by FESEM. The hMSC cultures onto fibers scaffolds were fixed with 4% paraformaldehyde. Then, the samples were washed with PBS and dehydrated with increasing concentrations of alcohol (25–50–75–90–100%, 5 min each) and air-dried. For fluorescence analyses, cells were preliminary incubated in phenol red-free medium with CellTracker Deep Red (Thermo Fisher scientific, Waltham, MA, USA) at 37 °C for 30 min. Then, cell culture was washed in phosphate bovine serum (PBS) and recovered for 1 h in complete medium. Lastly, cells were trypsinized, and placed onto the specimens for 24 h. After this period, the specimens were fixed with 4% paraformaldehyde and washed with PBS to evaluate the cell morphology via confocal microscopy (LSM510, Carl Zeiss, Germany).

## 3. Results and Discussion

Synthetic polymers such as PCL have been largely used for the fabrication of fibrous scaffolds for tissue engineering due to their biocompatibility [29] and mechanical stability ascribable to long degradation times [30]. However, recent works have discouraged the use of pure PCL scaffolds due to their limited capability to trigger cell adhesion mechanisms, ascribed to their intrinsic hydrophobic properties [31]. For this reason, natural proteins with innate biocompatibility can be variously combined with PCL fibers in order to provide selected biochemical signals to cells able to improve the biological response [32].

In this work, PCL was blended with three different proteins, namely gelatin, zein, and keratin, respectively, in order to compare the morphological and chemical/physical properties of fibers as a function of the peculiar contribution of the protein used. By an accurate control of the electrospinning process parameters, PCL/gelatin, PCL/zein, and PCL/keratin solutions were processed via electrospinning to form protein-rich fiber scaffolds. PCL solution was also processed to fabricate PCL fibers, used as a negative control.

**Figure 1:**
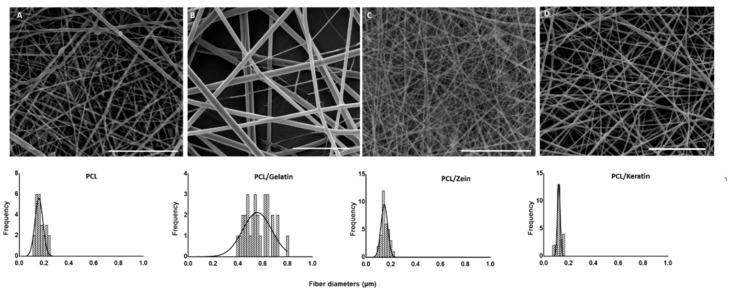
SEM images of PCL (**A**), PCL/gelatin (**B**), PCL/zein (**C**), and PCL/keratin (**D**) fibers (scale bar: 5 μm) and fiber diameter distribution via image elaboration (bottom).

As seen in Figure 1, random fibers obtained under optimized conditions were reported. Fibers from PCL solution showed the presence of some defects along the fiber body and average diameters of (0.171 ± 0.036) μm. In contrast, fibers from PCL/gelatin, PCL/zein, and PCL/keratin solutions were defect free and with diameters equal to 0.564 ± 0.102 μm, 0.153 ± 0.027 μm, and 0.124 ± 0.036 μm, respectively. Notably, narrow diameter distributions were detected in the presence of zein and keratin, while a wider diameter distribution was recorded in the case of PCL/gelatin fibers, probably due to problems in the homogeneous mixing of gelatin Type A in HFIP solution at the fixed concentration.

**Figure 2:**
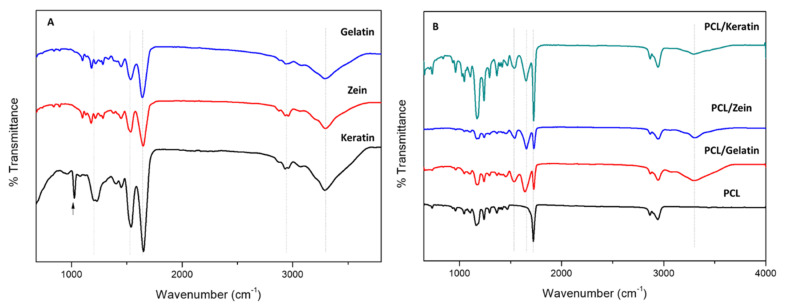
ATR-FTIR spectra of (**A**) gelatin, zein, and keratin nanofibers and (**B**) PCL/keratin, PCL/zein, and PCL/gelatin. Spectrum of net PCL nanofibers was reported as negative control.

Once the morphology of fibers was investigated, the efficient incorporation of the natural proteins into the fibers was confirmed via ATR-FTIR spectroscopy analyses (Figure 2). Spectra of natural proteins were reported as controls (Figure 2A) by using samples taken from pure protein solutions. The characteristic bands corresponding to amide I, amide II, and amide III at 1650 cm^−1^ (C=O stretching), 1540 cm^−1^ (N–H bending associated with C–N stretching), and 1240 cm^−1^ (C–N and N–H groups of bound amide); and amide A at 3286 cm^−1^ [33,34,35,36] were detected in all the spectra (Figure 2A). A broad band at 1195 cm^−1^ and a narrow peak at 1020 cm^−1^ were recognized in the case of the keratin spectrum (see the arrow), related, respectively, to S–O stretching vibrations of the Bunte salt residues produced during keratin extraction [33].

The same peaks could be recognized in the spectra of protein-rich electrospun fibers by overlapping with some peaks basically present in the PCL fibers. In particular, all the characteristic peaks of PCL were clearly remarked at 2949 cm^−1^ (asymmetric CH_2_ stretching), 2865 cm^−1^ (symmetric CH_2_ stretching), 1727 cm^−1^ (carbonyl stretching), 1293 cm^−1^ (C–O and C–C stretching), 1240 cm^−1^ (asymmetric C–O–C stretching), and 1170 cm^−1^ (symmetric C–O–C stretching) [37]. Independently upon the protein used, the spectra of the samples with proteins also showed the characteristic bands corresponding to amide I (1650 cm^−1^) and amide II (1530 cm^−1^) that are distinctive of the proteins, definitely confirming their presence into/onto the fibers.

**Figure 3:**
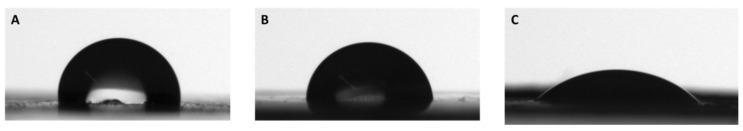
Water contact angle of bicomponent scaffolds of PCL/gelatin (**A**), PCL/zein (**B**), and PCL/keratin (**C**).

In Figure 3, water contact angle measurements were reported. We demonstrate that the hydrophobic behavior of PCL was reduced by the presence of the proteins; in particular, keratin greatly reduced the water contact angle of PCL, while zein contributed only to a slight reduction of hydrophobicity, and gelatin was close to PCL. In fact, pure PCL nanofibers have a water contact angle of 133° ± 2.3° [38], while PCL/gelatin, PCL/zein and PCL/keratin have water contact angles of 95.2° ± 1.2°, 76.3° ± 4.0°, and 44.3° ± 3.9°, respectively. The results can be explained taking into account both the intrinsic wettability of the polymers and the diameter distribution of the fibers. PCL/gelatin fibers had a wide diameter distribution, whilst PCL/zein, and PCL/keratin fibers had comparably narrow distributions. On the other hand, keratin have more hydrophilic side chain chemical groups (e.g., Bunte salts) that promoted a decrease in water contact angle, in comparison to zein, which is partially hydrophobic, being rich in hydrophobic and neutral amino acids as well as some sulfur-containing amino acids but lacking in polar/ionizable amino acids [39].

Hence, surface properties of fibers with different wettability were also tested in vitro in order to evaluate the different behavior of hMSCs in the presence of the different proteins.

**Figure 4:**
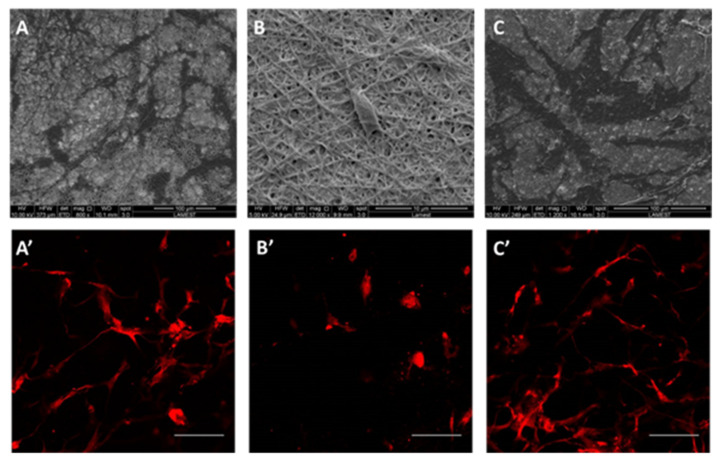
SEM images of hMSCs after 24 h in culture onto bicomponent scaffolds of PCL/gelatin (**A**), PCL/zein (**B**), and PCL/keratin (**C**). Confocal images of hMSCs cultured after 3 days onto bicomponent scaffolds (**A’**–**C’**), respectively (scale bar: 20 µm).

Cell morphology of hMSCs seeded onto scaffolds was investigated by SEM analysis, as reported in Figure 4A–C. After 24 h in culture, hMSCs were distributed onto the protein-rich fibers that promoted the formation of clusters. It was possible to distinguish elongated structures along fibers. Moreover, after 3 days in culture, hMSC interaction with protein-rich fibers was further investigated by confocal analysis (Figure 4A’–C’). In the presence of PCL/gelatin and PCL/keratin fibers, hMSC showed several projection of their cytoplasm, which corresponded to lamellipodia and greater cell density. In the case of PCL/zein fibers, cells were differently grown, forming isolated small clusters onto the scaffold surface, showing a rounded morphology and shorter cytoplasmatic projections as typically remarked in the case of low biological recognition of the surfaces.

**Figure 5:**
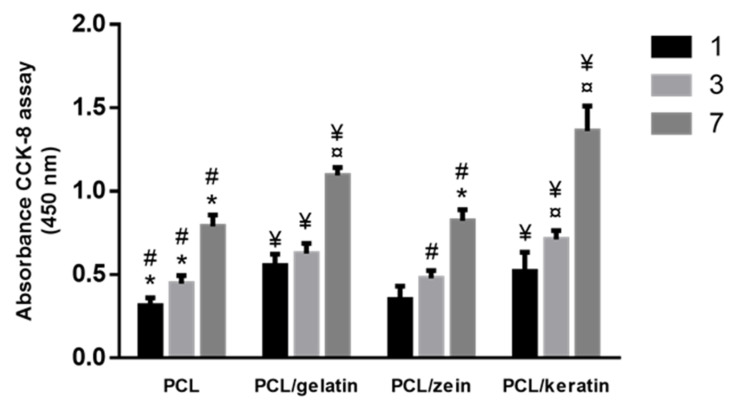
Study of hMSC viability onto protein-rich fibers until 7 days: ¥ denotes statistical significance PCL fibers; * denotes statistical significance difference compared to PCL/gelatin fibers; ¤ denotes statistical significance compared to PCL/zein fibers; # denotes statistical significance difference compared to PCL/keratin fibers (*p* < 0.05).

Moreover, in comparison with zein, gelatin is considered an excellent non-immunogenic alternative to the collagen, with the further advantage, with respect to the collagen, that it does not show any denaturation phenomena due to the interaction with the applied electric field during electrospinning process [40]. Recent studies have demonstrated that keratin and gelatin can influence similarly cell proliferation, due to their content of RGD-like and LDV-like adhesion motifs that improve the interface between cells and materials [41,42]. Accordingly, we have demonstrated that cell viability was enhanced in presence of gelatin and keratin proteins, while a significant reduction was recognized in the case of PCL/zein and PCL fibers. The viability of hMSCs onto all the protein-rich fibers is seen in Figure 5. Despite the good biocompatibility of zein, cell behavior was similar to PCL fibers (i.e., no significant difference), probably due to the less hydrophilic properties of zein in comparison with other natural proteins, in agreement with previous studies [43]. Contrariwise, a higher response of hMSCs was detected in the case of PCL/gelatin and PCL/keratin, where both morphological and chemical signals influenced cell behavior.

## 4. Conclusions

In this work, a comparative study of protein-rich scaffolds, fabricated via electrospinning, was carried out. PCL was blended with three different proteins (i.e., gelatin, keratin, and zein), to form fibrous scaffolds mimicking the architecture of the native ECM and providing selected biochemical moieties to improve the cell recognition and triggering in vitro biological response. We verified that the three different proteins used, due to their peculiar chemical features, can generate fibers with several differences in terms of fiber morphology, i.e., average diameters and distribution. Among them, keratin contributes to affect hydrophilicity of fiber surfaces and fiber diameter distribution, both influencing the in vitro response in terms of adhesion and hMSC proliferation. In agreement with previous works, these results confirm the successful use of keratin blended with PCL for the fabrication of biologically instructive scaffolds suitable for early in vitro studies. In perspective, keratin-rich fibers will be further investigated to optimize crosslinking strategies, based on the use of not toxic compounds, which are able to improve in vitro stability of proteins and, accordingly, biomechanical properties of fibers, in order to guarantee a long term biological response. This will address the use of keratin-rich fibers for in vitro interface applications, such as instructive scaffolds for interface tissue engineering or bio-functional coatings for biomedical devices.

## Figures and Tables

**Table 1:** Electrospinning parameters used to fabricate protein-rich fibers.

Polymer Solution	Voltage Applied (kV)	Flow Rate (mL/h)	Tip-to-Collector Distance (mm)
PCL	22	0.1	120
PCL/Gelatin	15	0.1	130
PCL/Zein	12	0.1	130
PCL/Keratin	25	0.1	120
